# Investigation on Morphology and Mechanical Properties of Rod Units in Lattice Structures Fabricated by Selective Laser Melting

**DOI:** 10.3390/ma14143994

**Published:** 2021-07-16

**Authors:** Chenchen Jing, Yanyan Zhu, Jie Wang, Feifan Wang, Jiping Lu, Changmeng Liu

**Affiliations:** 1 School of Mechanical Engineering, Beijing Institute of Technology, Beijing 100081, China; jingchenchen@live.com (C.J.); zhyy2913@bit.edu.cn (Y.Z.); jipinglu@bit.edu.cn (J.L.); 2 China Academy of Launch Vehicle Technology, Beijing Institute of Astronautical Systems Engineering, Beijing 100076, China; ruifangwang0@163.com (J.W.); wangifw@hotmail.com (F.W.)

**Keywords:** lattice structures, selective laser melting, Ti-6Al-4V, morphology, mechanical properties

## Abstract

Selective laser melting (SLM) fabrication of lattice structures has attracted considerable interest due to its many immanent advantages, such as high specific strength. A wide variety of lattice structures have been designed and fabricated. However, as a vital prerequisite for design optimization, a clear relation between the process constraint of SLM and the apparent properties of the fabricated lattice structure has received much less attention. Therefore, this work systematically investigates the characterization and preformation of rod units, which are the basic components of lattice structures, so as to evaluate the SLM manufacturability of lattice structures. A series of rod units with different inclination angles and diameters were fabricated by SLM. Their morphology and mechanical properties were measured by scanning electron microscope observation and a tensile test, respectively. The inclination angle was found to have significant effects on profile error and little effect on mechanical properties. The higher the inclination angle, the larger the profile error. The characteristic diameter had no significant correlation with profile errors and mechanical properties. Based on systematic studies, a formula is proposed to evaluate the cross-sectional area of the fabricated rod units and further estimate their load capacity. This has important implications for optimizing the design of lattice structures fabricated by SLM.

## 1. Introduction

In a wide variety of engineering applications, such as in the aerospace, military, automotive, and medical industries, the weight of parts has significant impacts on both functional performance and usage cost [1,2,3,4]. As a typical light weight structure, lattice structures continue to receive considerable interest, as they not only provide good mechanical properties (such as exceptional load bearing efficiency and customizable stiffness), but also possess some intriguing functionalities such as energy absorption, acoustic and elastic wave manipulation, etc. [5,6,7,8,9,10]. Traditional manufacturing methods of three-dimensional lattice structures include investment casting [11], deformation forming [12], woven/non-woven metal textiles [13], etc. [14,15]. These methods are complex, costly, have low material utilization efficiency [16], and it is difficult to fabricate very complicated lattice structures. This is where additive manufacturing, also known as 3D printing, comes into play. Additive manufacturing is a technique that fabricates a part layer by layer to form, theoretically, any complex geometries [17]. It provides unprecedented design freedom for fabricating lattice structures and creates much less waste [15]. Selective laser melting (SLM) is one of the most widely used additive manufacturing techniques [18,19]. The process of SLM fuses the powder selectively by laser power, layer by layer, according to the slicing of the parts. The powder, fully melted by laser power, will combine with the previous layer and form a dense part [20,21,22,23]. This method proposes an idea to fabricate complex lattice structures [24]. As for now, significant efforts have been made to use SLM to fabricate lattice structures. For example, Arabnejad et al. [25] proposed two high-strength stretch-dominated cell topologies, and fabricated high-strength porous biomaterials using SLM. Yan et al. designed a new cell type called the gyroid cellular lattice structure [5] and investigated its manufacturability and performance fabricated by SLM [26]. Al-Saedi et al. [27] fabricated a functionally graded lattice structure by SLM, which has a higher energy absorption capacity than a uniform lattice structure. 

On the other hand, SLM also has its own processing constraints. When one designs the lattice structure, such processing constraints must be carefully taken into account. This is the vital prerequisite for the design optimization of lattice structures fabricated by SLM. For example, SLM requires a support structure to achieve the required build geometry (for example, fabricating the cantilever structure). This support will be removed after manufacturing. The lattice structure is composed of a large number of cantilever rod units, and support cannot be added since it is inconvenient to remove and will greatly increase surface roughness [28]. This implies that not all build inclinations are permitted in lattice structures. The influence of the angle between the rod and the build direction on the lattice structure is negatively impacted by the principles of the SLM method. In addition, when one designs or predicts the properties of the lattice structure, the same material properties are generally used for all the rod elements in the lattice structure. However, it is well-known that SLM process can compromise the quality of some rod units. A layer-by-layer build-up process makes the parts become anisotropic, which leads to heterogenous mechanical properties in different directions [29,30,31]. If a clear relation between the process constraint of SLM and the apparent properties of the fabricated lattice structure is not built, one cannot achieve the integrated structure-functionality-fabrication design.

Although a few reports have been presented on this subject, the discussions, especially for mechanical properties, are generally based on the analysis of the whole lattice structure. The complex anisotropic properties of the structure make it difficult to isolate the individual relative contribution of each main unit rod on the overall mechanical response. For example, Leary et al. [32] discussed the manufactural feature size and roughness of structure elements in several lattice structures, such as body centered cubic (BCC), face centered cubic (FCC), body centered cubic with vertical structures (BCCZ), etc. They presented the failure mechanisms and energy absorption characteristics of these lattice structures. Kadirgama et al. [33] investigated Young’s modulus, surface roughness, and yield stress of lattice structures which involved four processing parameters (strut size, struct shape, unit cell, and porosity). They found that porosity has a significant influence on both Young’s modulus and compressive strength. 

Different from these previous works, this work focuses on the unit rod of the lattice structure. It is therefore hoped that the studies will help us to evaluate the separate maneuverability and typical properties of the unit rod, and guide the optimal design of lattice structures. Ti-6Al-4V is chosen due to its wide application. Multiple sets of Ti-6Al-4V rod unit structure samples were designed and manufactured by SLM using different inclination angles and diameters. One group did not have support, in order to observe the forming performance of the rod units. The other group uses partial support to test the mechanical properties of the rod. The inclination angle and feature diameter represent the two main features during the design of the rod unit of lattice structures. Therefore, their coupling effects are investigated. The quality and the mechanical response of the fabricated samples are discussed. The error fit formula and the cross-sectional area calculation are presented to estimate the load capacity of the rod unit. The result can serve as a reference for future lattice structure designing.

## 2. Materials and Methods

### 2.1. Sample Design

**Figure 1:**
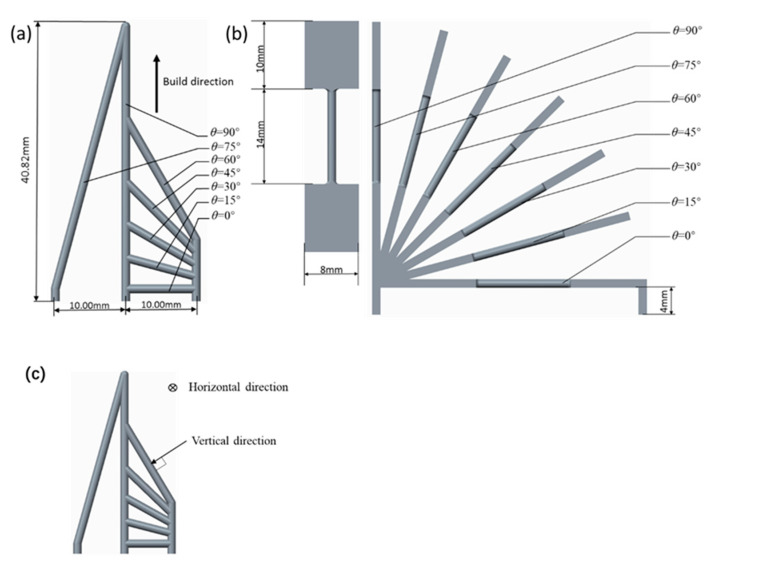
Detail of the sample structure: (**a**) RA sample used for SEM observing; (**b**) RB sample used for tensile test; and (**c**) the view of horizontal direction and vertical direction.

**Figure 2:**
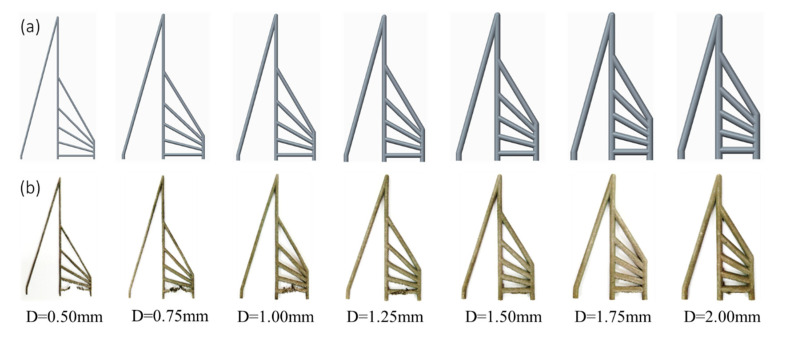
RA sample: (**a**) the CAD model of RA sample for rod diameter of 0.50–2.00 mm in increments of 0.25 mm and (**b**) RA Sample manufactured by SLM.

**Figure 3:**
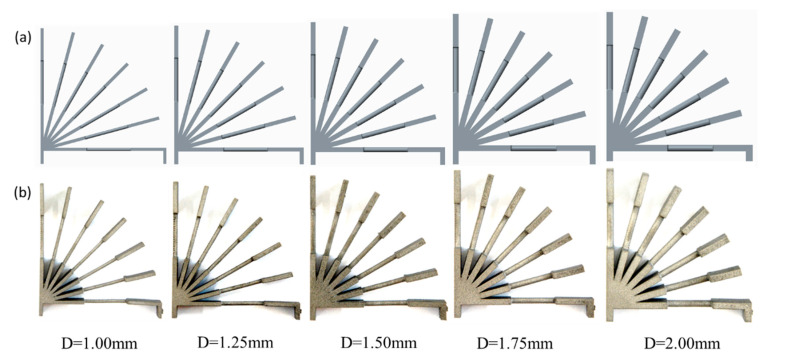
RB sample: (**a**) the CAD model of RB sample for rod diameter of 1.00–2.00 mm in increments of 0.25 mm and (**b**) the RB Sample manufactured by SLM.

In this research, two kinds of rod unit structures were designed. One group of the samples was a pure rod unit referred to as ‘RA’, which was used to study the coupled effect of the build direction and the rod diameter on the forming quality of the rod. This group of samples had 49 sets of setups consisting of different combinations of the inclination angle θ (0–90°, in 15° increments) and diameter D (0.50–2.00 mm, in increments of 0.25 mm). The definition of the inclination angle θ is marked in Figure 1a. The height of the specimens was 40.82 mm, and the distance between the vertical rods was 10 mm. The corresponding CAD models of RA samples are shown in Figure 2a. The other group was designed for mechanical testing. They are referred to as ‘RB’ consisting of a rod sandwiched by two plates, as shown in Figure 1b. The rod length was 14 mm, the plate was 10 mm in length, 8 mm in width, and the thickness of the plate was the same as the rod diameter. The horizontal rod was 4 mm higher than the substrate. As in the Table 1 list, the 35 sets tensile specimens were set. The rod diameter D was set from 1.00 to 2.00 mm with a 0.25 mm increase, and the inclination angle was set 0 degree to 90° with a 15° increase (as illustrated in Figure 1b). This set number was lower than that of group 1, because some unmanufacturable rods were excluded. The CAD models of the tensile specimens are shown in Figure 3a. The tensile specimens were cut by electrical discharge machining after being manufactured completely.

### 2.2. Sample Manufacturing

Titanium (Ti) alloys have high specific strength and great creep and corrosion resistance [34]. They are widely used in the aerospace field, military field, biomaterial field, etc. [30,35,36]. Therefore, Ti-6Al-4V was chosen and fabricated. SLM manufacturing was performed using EOSINT M 280 (EOS GmbH, Munich, Germany). The powder was provided by EOS GmbH, and the averaged powder diameter was 36 µm. The optimal fabrication process parameters for Ti-6Al-4V were chosen as shown in Table 2. Different from printing RA samples without any support, the RB samples exerted the necessary support for the cases of small inclination angle.

### 2.3. Macrostructure Observation and Tensile Tests

Once the manufacturing of the samples had been finished, the diameter of the rod units in the RA sample was measured using SEM. The morphologies of the rod units were studied from the horizontal, vertical, and cross-section directions, respectively, as indicated in Figure 1c. The rod was cut with electrical discharge machining for easy visualization. To prevent the adhesion of powder and other impurities on the observed results, the samples were repeatedly cleaned before observation. Ten random points were measured in each sample, and their average values were used.

The RB samples were cut into individual tensile specimens by electrical discharge machining. Tensile tests were carried on INSTRON 5966 (Instron, Boston, MA, USA). Three samples were used in each group. The tensile tests were conducted according to ASTME-E8/E8M-15a [37].

## 3. Results & Discussion

### 3.1. Macrostructure Observation of RA Samples

To investigate the coupled effect of inclination angle and design diameter on the quality and morphology of rod units, RA samples without support were designed and fabricated. The manufactured RA samples are shown in Figure 2b. Since no support was applied, the rod element in the horizontal direction could not be formed when the rod was thin (0.50–1.00 mm). When the diameter increased, the horizontal rod element was gradually stabilized. With the increase of inclination angle, the rod element was more stable and the surface roughness was smaller. 

**Figure 4:**
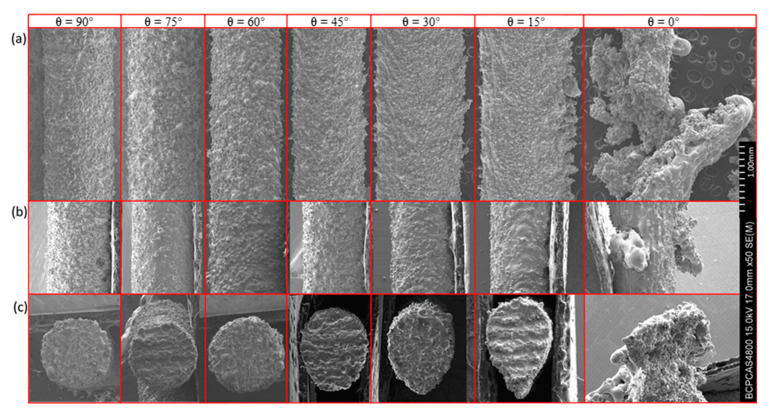
RA sample SEM image for design diameter of 1 mm and the inclination angles of 90–0° in 15° decrements: (**a**) taken from the horizontal direction; (**b**) taken from a vertical direction; and (**c**) taken from the cross section.

In order to observe the morphology of the sample clearly, Figure 4 shows the SEM images of the rod elements. It reflects the change of the morphology due to inclination angle. From left to right, the inclination angle changed from 90 to 0°, while the diameter *D* was kept as 1.00 mm. Note that the building direction of SLM is from right to left, and from bottom to top. Figure 4a is a photograph taken from the horizontal direction (longitudinal profile of the rod as shown in Figure 1c; Figure 4b is a photograph taken from a vertical direction (lateral profile of the rod) with the displayed surface as an upper surface; and Figure 4c is taken from the cross section of the rod. These three directions can well describe the morphology of the sample and reveal the influence of inclination angle on morphology. It can be seen clearly that with the decrease of the inclination angle, the longitudinal dimension gradually increased, and the lateral size only changed a little. When the inclination angle was greater than 45°, the shape of the cross section was close to a circle. When the inclination was smaller than 45°, the top of the cross section was kept circular while the bottom was a conical shape. In addition, the taper increased with the decrease of inclination.

**Figure 5:**
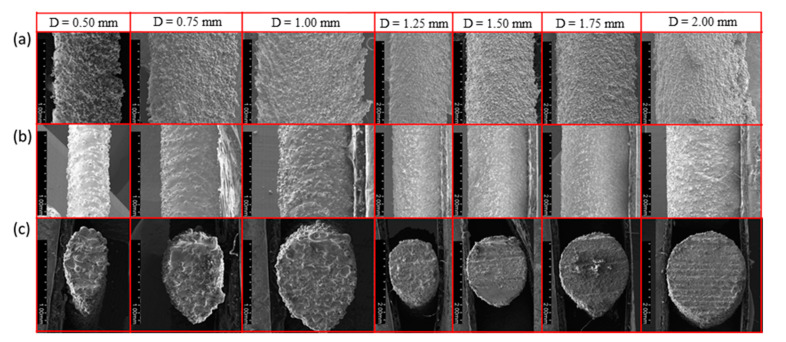
RA sample SEM image for inclination angles of 30° and the design diameters of 0.25–2.00 mm in increments of 0.25 mm: (**a**) taken from the horizontal direction; (**b**) taken from a vertical direction; and (**c**) taken from the cross section.

Figure 5 shows the SEM images of rod elements with different diameters at the same inclination angle (*θ* = 30°). From Figure 5a,b, the downward surface of the rod is rougher than the upward one, because of the preferential particle adhesion. As the diameter increased, the changes in Figure 5a,b, were not obvious. It indicates that the amount of metal droplets sinking is constant for the same inclination angle. However, it can be seen that with the increase of the diameter of the rod element, the shape of the cross section is close to a circle, which indicates that the shape error decreased gradually.

**Figure 6:**
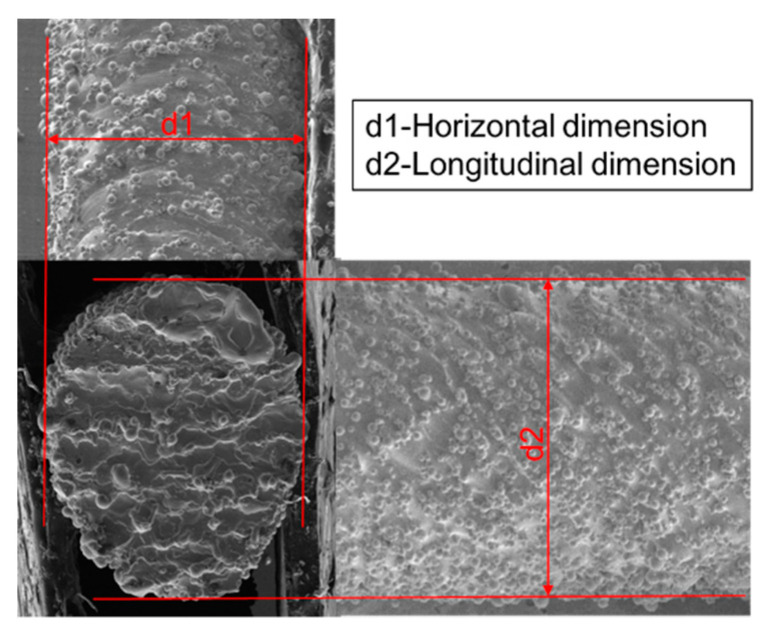
Schematic view of the rod diameters size measurement.

**Figure 7:**
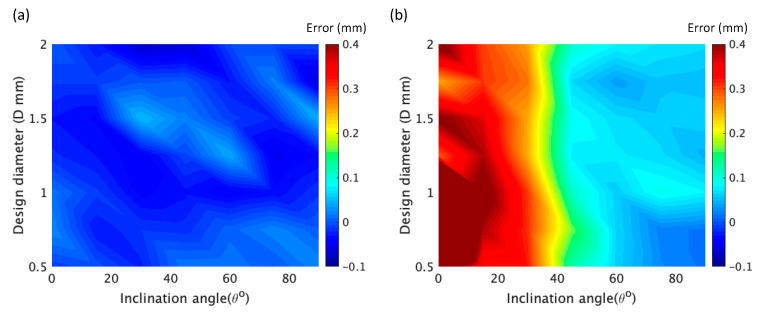
(**a**) Coupled influence of design diameter *D* and inclination angle *θ* on error *ε* of the horizontal dimension d1 of the rod unit. (**b**) Coupled influence of *D* and *θ* on *ε* of the longitudinal dimension d2 of the rod unit; d1 and d2 are indicated in Figure 6.

**Figure 8:**
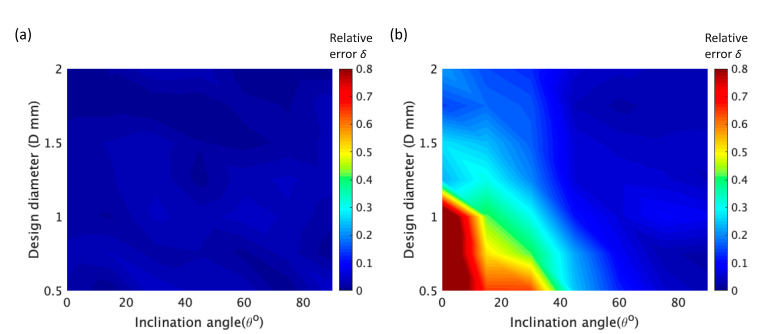
(**a**) Coupled influence of design diameter *D* and inclination angle *θ* on relative error *δ* of the horizontal dimension d1 of the rod unit. (**b**) Coupled influence of *D* and *θ* on *δ* of the longitudinal dimension d2 of the rod unit; d1 and d2 are indicated in Figure 6.

In order to further qualify the shape error of the rod element, the lateral and longitudinal dimensions of the rod were measured. Figure 6 schematically shows the horizontal dimension d1 and longitudinal dimension d2 of the rod, along the building direction. Diameters of all the rods were measured in the direction perpendicular to their axis. For every rod unit, 10 values of the rod diameters were measured and the average value, *d*, could be readily obtained. The error was calculated as ε=d−D. Then, the relative error *δ* was evaluated as $\delta =\frac{\left|d-D\right|}{D}$,where *d* is the measured diameter, and *D* is the design diameter. The average diameter and its standard deviation are given in Table 3. The relative error is listed in Table 4. Figure 7 and Figure 8 show the coupled effect of rod diameter and inclination angle on the shape error of the rod element.

By synthesizing these data, it can be seen that the error of the longitudinal dimension is much higher than that of the horizontal dimension. This means that the error along the bottom-to-up building direction is higher than that along the left-to-right building direction. Along the left-to-right building direction, the average relative error of the rod element was below 0.05, according to Figure 8a. However, along the bottom-to-up building direction, the error become acceptable (smaller than 0.1 mm) only when the inclination angle was higher than 45°, which is insensitive to the design diameter, as shown in Figure 7b. With the increase of the design diameter, the relative error decreased (see Figure 8b).

How should we understand such shape error? Since there is only powder support in the first few layers of the fabricated rod, the laser melts more powder under the bottom layer which eventually adheres to the rod. Moreover, due to the instability of the powder support, the melted metal droplets will subside. The depression will fill with more powder with the ongoing process of the spreading powder. Both of the processes above can increase the rod diameter along the bottom-to-up building direction. With the increase of the inclination angle, these effects are gradually reduced and the error decreases. Since the degree of laser melting and the degree of metal droplet subsidence are fixed, it does not change with changes of rod diameter. Thus, when the rod diameter is increased, the relative error is reduced.

### 3.2. Tensile Test of RB Samples

**Figure 9:**
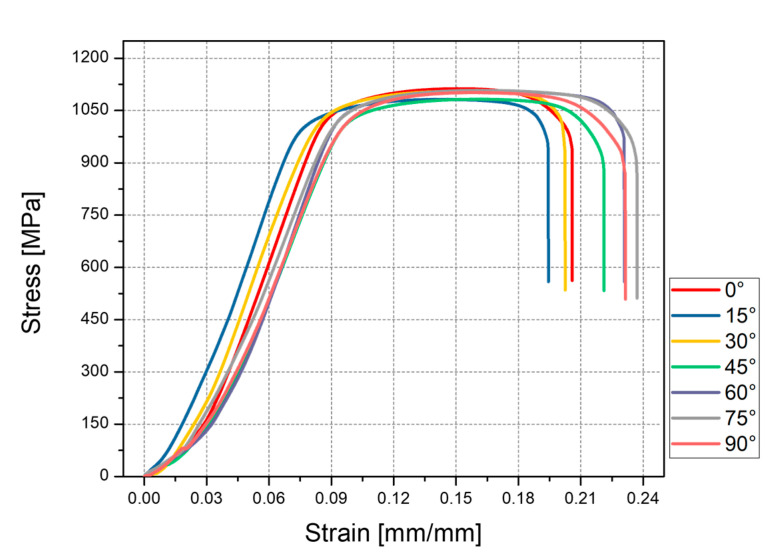
Stress–strain response of the RB sample with diameter of 1.75 mm.

**Figure 10:**
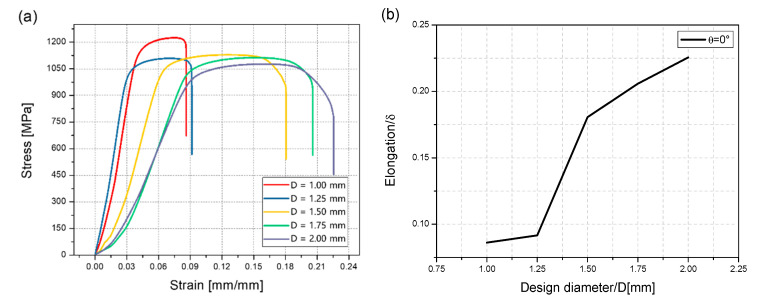
(**a**) Stress–strain response of the RB sample with inclination angle of 0°. (**b**) Elongation–Design diameter response of the RB sample with inclination angle of 0°.

**Figure 11:**
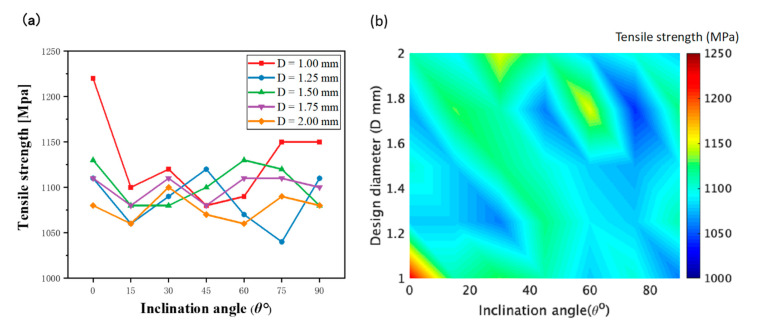
(**a**) Tensile strength-inclination angle curves with different diameter. (**b**) Coupled influence of the inclination angle *θ* and design diameter *D* on the tensile strength of rod unit.

**Figure 12:**
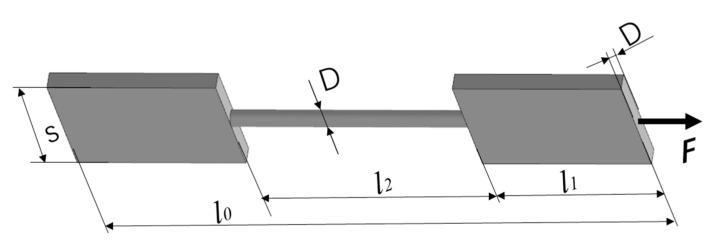
Schematic diagram of the tensile sample.

To explore the anisotropic mechanical properties of the rod units, the RB samples with partial support were designed and manufactured. The fabricated RB samples are shown in Figure 3b. Necessary support was added when printing in order to ensure consistent cross-sectional area of the samples for tensile tests. The rod unit diameter was found to be almost uniform, which can be seen to some extend from Figure 3b. All of the samples were cut into individual tensile specimens by electrical discharge machining. The stress–strain curves of the rod elements at different inclination angles when the design diameter was 1.75 mm are shown in Figure 9. As we can see, the little influence has reflected the inclination angle of rod on mechanical properties. This weak influence of inclination angle is also observed for rod elements with other diameters. The stress–strain curves of the rod elements with different diameters, and the inclination angle the same as 0° are shown in Figure 10. It can be seen that the tensile strength exhibits weak size effect. Namely, the smaller the diameter the higher the tensile strength. Such week size dependence is only observed when the inclination angle is smaller than about 20°, as shown in Figure 11b. The change of the tensile strength is not significant. On the other hand, Figure 10b shows that the elongation increases with the increase of the rod diameter. This is expected since a smaller diameter corresponds to larger aspect ratios of the rod unit. Actually, the different slopes of elongation are caused by the different equivalent modulus with the rod diameter changed. Figure 12 shows the schematic diagram of the tensile sample. The *D* is the diameter of the rod, and *l*_2_ is the rod’s length. *l*_1_ is the plate length, *s* is the plate width, and the thickness is equal to *D*. The stretching force is ***F***. Combined with Equations (1)–(3), the equivalent modulus *E_e_* can be determined as Equation (4). Δ*l* is the stretched length, *ε_e_* is the equivalent strain, σ*_e_* is the equivalent stress, and *E* is the Young’s modulus. As we can see in Equation (4), as the diameter increases, the equivalent modulus will decrease. Table 5 summarizes the tensile strength of each rod and is shown in Figure 11. The tensile strength of all rods is about 1100 MPa. It can be concluded that the diameter and inclination angle have no significant impacts on the mechanical properties of the rod. The smaller rod with lower inclination angle exhibits a little higher tensile strength.

(1) $$\Delta l=\frac{F}{\left(Ds\right)E}l_{1}\times 2+\frac{F}{\frac{1}{4}\pi D^{2}E}l_{2}$$

(2) $$\varepsilon_{e}=\frac{\Delta l}{l_{2}}$$

(3) $$\sigma_{e}=\frac{F}{\frac{1}{4}\pi D^{2}}$$

(4) $$E_{e}=\frac{\sigma_{e}}{\varepsilon_{e}}=\frac{E}{\frac{\pi Dl_{1}}{2sl_{2}}+1}$$

The mechanical property of Ti-6Al-4V is directly determined by the lamellar α microstructure, the β grain orientation, and the β grain size [38,39,40]. In the SLM fabricating component, the different orientation of the β grain is the key to influencing the anisotropy of mechanical properties [40]. It leads to a small size of the β grains and the lamellar α microstructure for tiny molten pool and high cooling rate in the small rod elements of lattice structures. The tensile strength of the rod unit is up to 1100 MPa, which is close to the data reported before [31]. The effect of grain orientation is relatively weak. Therefore, there is no obvious anisotropy of the mechanical properties in the tensile test. It is good news for designers that the anisotropy of the tensile strength can be ignored when designing lattice structures for the considered ranges. 

### 3.3. Error Fitting and Area Calculation

**Figure 13:**
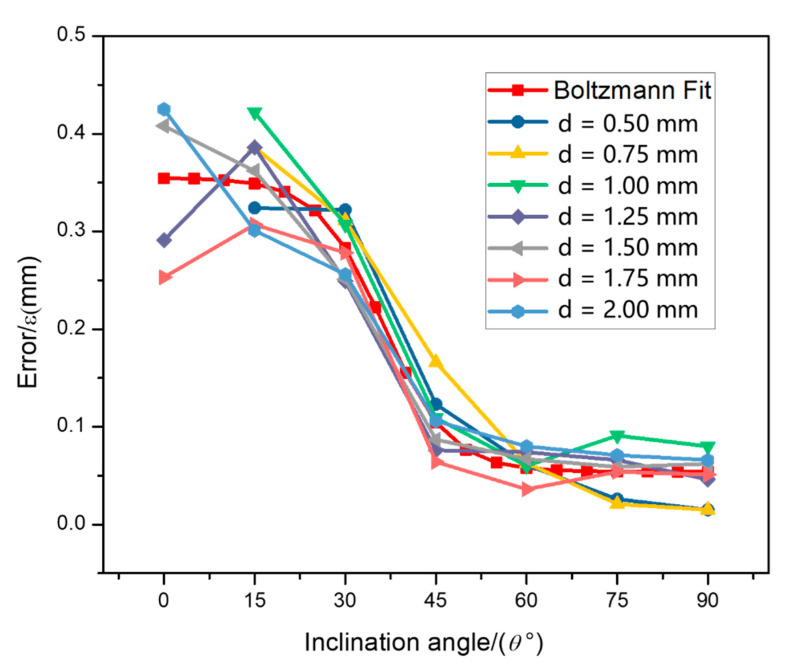
Comparison of longitudinal error between Boltzmann curve fitting and actual rod unit.

The previous section indicates that the SLM process has no obvious influence on the mechanical properties of the rod element if uniform cross-section is used. Thus, the cross-sectional area is the main factor which affects the load capacity of the rod element. To better guide the lattice structure design, it will be great to obtain the relation between the cross-sectional area and the inclination angle. At first, nonlinear fitting of the longitudinal error is carried out and the Boltzmann function [41] can be fitted well. The formula is as follows:

(5) $$\varepsilon \left(\theta \right)=0.050+\frac{0.33}{1+e^{\frac{\theta -34}{6.1}}}$$

where *ε*(*θ*) refers to the longitudinal error when the inclination angle is *θ*. The R-Square (COD) is 0.93 and the Adj. R-Square is 0.92. Figure 13 shows the comparison of longitudinal error between Boltzmann curve fitting and the actual rod unit. It can be seen that actual values agree very well with the fitted values.

**Figure 14:**
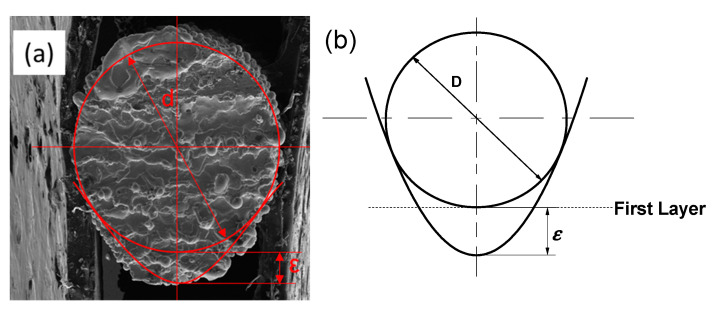
(**a**) Cross section of rod unit. (**b**) Geometric model of cross section.

Moreover, the cross-sectional area can be approximated by the diameter *D* and longitudinal error *ε*. The cross section of the rod can be seen surrounded by a circle and a parabola (Figure 14a). It can be approximated using a model of Figure 14b. The parabola is tangent to the circle and its vertex is *ε* from the bottom of the circle. The cross-sectional area can be determined as:

(6) $$A=\frac{4}{3}\sqrt{r^{2}-\frac{1}{4a^{2}}}\left(b-\frac{1}{2a}\right)+r^{2}\left(\pi -{cos}^{-1}\left(\frac{1}{2ar}\right)\right)+\frac{1}{2a}\sqrt{r^{2}-\frac{1}{4a^{2}}}$$

(7) $$a=\frac{\sqrt{b^{2}-r^{2}}+b}{2r^{2}}$$

**Figure 15:**
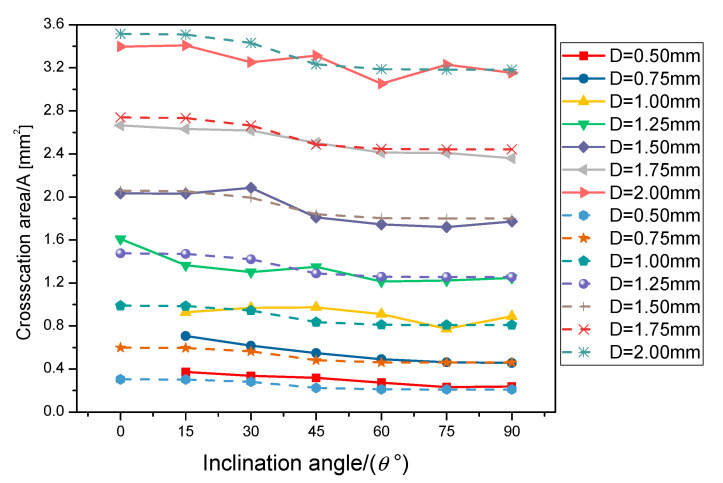
Comparison of measured values and calculated values. Solid line represents the measured value and dash line represents calculated value.

(8) b=r+ε

where *A* is the cross-sectional area, *ε* is the longitudinal error, and *r* is the radius of the rod. The cross-sectional area of the rod can be calculated from the diameter and inclination angle, in conjunction with Equations (5)–(8). In order to verify the accuracy of the formula, the cross-sectional area was measured by taking a rod unit from each of the RA samples and comparing it with the calculated cross-sectional area (Figure 15). The solid line in the figure represents the measured value and the dotted line represents the calculated value. It can be seen that the measured value and the calculated value are very close.

## 4. Conclusions

To guide the optimal design of lattice structures, a clear relation between the design parameter and the performance of the rod units of the lattice structure must be obtained. In this work, the coupling effects of the two most widely-used design parameters (i.e., the inclination angle and feature diameter) were systematically studied. Two groups of samples were fabricated using SLM, both with and without support. Through morphological observation and mechanical testing, the following conclusions were obtained:

(1) When fabricating lattice structures using SLM, there is generally no support because it is difficult to remove when constrained by limited access. In the case of there being no support, the inclination angle has a significant influence on the forming quality of the rod. With the increase of the inclination angle, the morphological error of the rod element is gradually reduced. When the inclination angle is below 30°, the error along the bottom-to-up direction is large (more than 0.2 mm). The error decreases rapidly when the inclination angle is 45°. The diameter of the rod has little effect on the profile error. However, with the increase of design diameter, the relative error decreases gradually.
(2) To fabricate tensile test samples, some additional support was used during SLM fabrication. The rod unit exhibited good mechanical properties and the tensile strength was up to 1100 MPa. The inclination angle and the diameter of the rod element have a limited influence on the mechanical properties of the rod, which implies that it is reasonable to use isotropic tensile strength value when designing the lattice structure.
(3) The error fitting formula and cross-sectional area calculation formula of the rod units were proposed. The former can determine the longitudinal error according to the inclination angle. The later can estimate the cross-sectional area of rod unit after fabrication.
(4) In summary, this study shows that the inclination of rod element has a great influence on the shape of the rod, while the mechanical properties of the rod are not anisotropic due to the angle change. The formulas proposed in Section 3.3 can be used to predict the cross-sectional area of rod element after fabrication. The load capacity of the rod can be estimated accordingly, which provides a reference for lattice structure design.

## Figures and Tables

**Table 1:** The design parameter of the sample.

Inclination Angle *θ* (°)	Design Diameter (D/mm)						
	RA Sample		RA & RB Sample				
**0**	0.50	0.75	1.00	1.25	1.50	1.75	2.00
**15**	0.50	0.75	1.00	1.25	1.50	1.75	2.00
**30**	0.50	0.75	1.00	1.25	1.50	1.75	2.00
**45**	0.50	0.75	1.00	1.25	1.50	1.75	2.00
**60**	0.50	0.75	1.00	1.25	1.50	1.75	2.00
**75**	0.50	0.75	1.00	1.25	1.50	1.75	2.00
**90**	0.50	0.75	1.00	1.25	1.50	1.75	2.00

**Table 2:** Processing parameters for the SLM-fabricated Ti-6Al-4V.

Deposition Parameters	Value
Laser power	260 W
Laser spot size	80 μm
Layer thickness	30 μm
Scanning speed	1200 mm/s
Hatch distance	40 μm

**Table 3:** The average diameter of the rod unit (mm)and standard deviation (in the brackets).

Design Diameter *D* (mm)		Inclination Angle *θ* (°)						
		0	15	30	45	60	75	90
0.50	d1	0.489 (5.284)	0.498 (2.507)	0.476 (0.946)	0.516 (0.567)	0.517 (2.565)	0.501 (1.315)	0.524 (1.953)
	d2	-	0.824 (3.055)	0.822 (0.775)	0.623 (1.431)	0.561 (0.133)	0.526 (0.678)	0.515 (0.856)
0.75	d1	0.774 (2.030)	0.721 (0.992)	0.735 (1.203)	0.732 (0.451)	0.756 (1.335)	0.773 (1.002)	0.753 (1.774)
	d2	-	1.136 (2.556)	1.061 (0.171)	0.916 (0.509)	0.813 (0.758)	0.771 (1.334)	0.765 (0.117)
1.00	d1	1.014 (4.524)	0.988 (1.502)	0.960 (0.425)	0.968 (0.247)	0.956 (0.623)	0.965 (0.512)	0.984 (0.312)
	d2	-	1.422 (2.007)	1.307 (3.211)	1.109 (1.217)	1.060 (0.327)	1.091 (0.376)	1.080 (0.248)
1.25	d1	1.230 (0.134)	1.220 (0.795)	1.206 (0.972)	1.241 (0.444)	1.291 (1.266)	1.200 (0.994)	1.213 (0.754)
	d2	1.541 (4.031)	1.636 (6.054)	1.499 (1.675)	1.326 (1.136)	1.324 (1.101)	1.316 (0.400)	1.296 (0.669)
1.50	d1	1.470 (1.011)	1.461 (0.484)	1.549 (0.645)	1.520 (0.909)	1.489 (2.802)	1.477 (1.712)	1.544 (1.599)
	d2	1.908 (5.124)	1.862 (3.957)	1.751 (4.218)	1.587 (0.840)	1.567 (0.383)	1.559 (0.643)	1.562 (0.567)
1.75	d1	1.738 (0.354)	1.738 (1.603)	1.750 (0.827)	1.752 (0.667)	1.720 (0.960)	1.770 (1.540)	1.689 (1.215)
	d2	2.003 (9.033)	2.057 (4.892)	2.028 (2.516)	1.814 (0.575)	1.786 (0.087)	1.804 (1.773)	1.801 (0.419)
2.00	d1	2.000 (1.157)	1.972 (0.213)	1.929 (1.102)	1.936 (1.811)	1.993 (0.607)	1.993 (0.802)	1.993 (1.558)
	d2	2.425 (7.274)	2.301 (0.611)	2.256 (2.989)	2.106 (0.837)	2.080 (0.746)	2.071 (1.236)	2.066 (0.179)

**Table 4:** The relative error of the rod unit.

Design Diameter *D* (mm)		Inclination Angle *θ* (°)						
		0	15	30	45	60	75	90
0.50	d1	0.022	0.004	0.048	0.032	0.034	0.002	0.048
	d2	-	0.647	0.645	0.246	0.121	0.053	0.030
0.75	d1	0.032	0.038	0.020	0.024	0.009	0.031	0.004
	d2	\	0.515	0.416	0.221	0.084	0.028	0.020
1.00	d1	0.014	0.0120	0.040	0.032	0.044	0.035	0.017
	d2	-	0.422	0.307	0.109	0.060	0.091	0.081
1.25	d1	0.016	0.024	0.035	0.007	0.033	0.040	0.030
	d2	0.233	0.309	0.199	0.061	0.059	0.053	0.037
1.50	d1	0.020	0.026	0.033	0.013	0.007	0.016	0.029
	d2	0.272	0.241	0.168	0.058	0.044	0.039	0.041
1.75	d1	0.007	0.007	0.000	0.001	0.017	0.012	0.037
	d2	0.145	0.175	0.159	0.037	0.021	0.031	0.029
2.00	d1	0.000	0.014	0.036	0.032	0.003	0.004	0.003
	d2	0.213	0.151	0.128	0.053	0.040	0.035	0.033

**Table 5:** Tensile strength of RB sample (MPa).

Inclination Angle *θ* (°)	Design Diameter *D* (mm)				
	1.00	1.25	1.50	1.75	2.00
0	1220	1110	1130	1110	1080
15	1100	1060	1080	1080	1060
30	1120	1090	1080	1110	1100
45	1080	1120	1100	1080	1070
60	1090	1070	1130	1110	1060
75	1150	1040	1120	1110	1090
90	1150	1110	1080	1100	1080

## Data Availability

Data sharing is not applicable to this article.
